# EANM procedure guidelines for brain PET imaging using [^18^F]FDG, version 3

**DOI:** 10.1007/s00259-021-05603-w

**Published:** 2021-12-09

**Authors:** Eric Guedj, Andrea Varrone, Ronald Boellaard, Nathalie L. Albert, Henryk Barthel, Bart van Berckel, Matthias Brendel, Diego Cecchin, Ozgul Ekmekcioglu, Valentina Garibotto, Adriaan A. Lammertsma, Ian Law, Iván Peñuelas, Franck Semah, Tatjana Traub-Weidinger, Elsmarieke van de Giessen, Donatienne Van Weehaeghe, Silvia Morbelli

**Affiliations:** 1 APHM, CNRS, Centrale Marseille, Institut Fresnel, Timone Hospital, CERIMED, Nuclear Medicine Department, Aix Marseille Univ, Marseille, France; 2 Service Central de Biophysique et Médecine Nucléaire, Hôpital de la Timone, 264 rue Saint Pierre, 13005 Marseille, France; 3 Department of Clinical Neuroscience, Centre for Psychiatry Research, Karolinska Institutet and Stockholm Healthcare Services, Stockholm, Sweden; 4 Department of Radiology and Nuclear Medicine, Amsterdam UMC, Location VUmc, Amsterdam, The Netherlands; 5 Nuclear Medicine and Molecular Imaging, University Medical Center Groningen, University of Groningen, Groningen, The Netherlands; 6 Department of Nuclear Medicine, Ludwig Maximilians-University of Munich, Munich, Germany; 7 Department of Nuclear Medicine, Leipzig University, Leipzig, Germany; 8 German Centre of Neurodegenerative Diseases (DZNE), Site Munich, Bonn, Germany; 9 Nuclear Medicine Unit, Department of Medicine – DIMED, University of Padua, Padua, Italy; 10 Sisli Hamidiye Etfal Education and Research Hospital, Nuclear Medicine Dept., University of Health Sciences, Istanbul, Turkey; 11 NIMTLab, Faculty of Medicine, Geneva University, Geneva, Switzerland; 12 Division of Nuclear Medicine and Molecular Imaging, Geneva University Hospitals, Geneva, Switzerland; 13 Department of Clinical Physiology, Nuclear Medicine and PET, Rigshospitalet, University of Copenhagen, Copenhagen, Denmark; 14 Department of Nuclear Medicine, Clinica Universidad de Navarra, IdiSNA, University of Navarra, Pamplona, Spain; 15 Nuclear Medicine Department, University Hospital, Lille, France; 16 Division of Nuclear Medicine, Department of Biomedical Imaging and Image-guided Therapy, Medical University of Vienna, Vienna, Austria; 17 Radiology and Nuclear Medicine, Amsterdam UMC, Location AMC, Meibergdreef 9, Amsterdam, The Netherlands; 18 Nuclear Medicine and Molecular Imaging, University Hospitals Leuven, 3000 Leuven, Belgium; 19 IRCCS Ospedale Policlinico San Martino, Genoa, Italy; 20 Nuclear Medicine Unit, Department of Health Sciences (DISSAL), University of Genoa, Genoa, Italy

**Keywords:** PET, Metabolism, Glucose, Dementia, Movement disorders, Epilepsy, Encephalitis, Psychiatry, Oncology, Glioma, Lymphoma

## Abstract

The present procedural guidelines summarize the current views of the EANM Neuro-Imaging Committee (NIC). The purpose of these guidelines is to assist nuclear medicine practitioners in making recommendations, performing, interpreting, and reporting results of [^18^F]FDG-PET imaging of the brain. The aim is to help achieve a high-quality standard of [^18^F]FDG brain imaging and to further increase the diagnostic impact of this technique in neurological, neurosurgical, and psychiatric practice. The present document replaces a former version of the guidelines that have been published in 2009. These new guidelines include an update in the light of advances in PET technology such as the introduction of digital PET and hybrid PET/MR systems, advances in individual PET semiquantitative analysis, and current broadening clinical indications (e.g., for encephalitis and brain lymphoma). Further insight has also become available about hyperglycemia effects in patients who undergo brain [^18^F]FDG-PET. Accordingly, the patient preparation procedure has been updated. Finally, most typical brain patterns of metabolic changes are summarized for neurodegenerative diseases. The present guidelines are specifically intended to present information related to the European practice. The information provided should be taken in the context of local conditions and regulations.

## Preamble

The European Association of Nuclear Medicine (EANM) is a professional nonprofit medical association that facilitates communication worldwide among individuals pursuing clinical and research excellence in nuclear medicine. The EANM was founded in 1985. These guidelines are intended to assist practitioners in providing appropriate nuclear medicine care for patients. They are not inflexible rules or requirements of practice and are not intended, nor should they be used, to establish a legal standard of care. The ultimate judgment regarding the propriety of any specific procedure or course of action must be made by medical professionals considering the unique circumstances of each case. Thus, there is no implication that an approach differing from the guidelines, standing alone, is below the standard of care. On the contrary, a conscientious practitioner may responsibly adopt a course of action different from that set out in the guidelines when, in the reasonable judgment of the practitioner, such course of action is indicated by the condition of the patient, limitations of available resources, or advances in knowledge or technology after the publication of the guidelines. The practice of medicine involves not only the science but also the art of dealing with the prevention, diagnosis, and treatment of disease. The variety and complexity of human conditions make it impossible to always reach the most appropriate diagnosis or to predict with certainty a particular response to treatment. Therefore, it should be recognized that adherence to these guidelines will not ensure an accurate diagnosis or a successful outcome. All that should be expected is that the practitioner will follow a reasonable course of action based on current knowledge, available resources, and the needs of the patient to deliver effective and safe medical care. The sole purpose of these guidelines is to assist practitioners in achieving this objective.

## Introduction

Approximately 95% of adenosine triphosphate (ATP) required for brain function is provided by glucose metabolism. The cerebral glucose metabolism is closely linked to neuronal and synaptic function in physiological conditions. During brain diseases, neuronal and synaptic changes consequently lead to glucose metabolic alterations. 2-[^18^F]fluoro-2-deoxy-D-glucose ([^18^F]FDG) is used with PET to image regional cerebral glucose consumption. [^18^F]FDG accumulates in brain tissue according to facilitated transport and hexokinase-mediated phosphorylation. The uptake of [^18^F]FDG also depends on the consumption of glucose by the astrocytes in interaction with neuronal function [1], on the transport mediated by the glucose transporter 1 (GLUT-1) across the blood-brain barrier [2], and possibly during pathological inflammatory conditions on microglial activation states [3]. Currently, [^18^F]FDG-PET, which is widely available in Europe, is the most accurate in vivo method for the investigation of regional human brain metabolism in clinical settings. Its clinical use is considered as established for several indications in neurology and neurosurgery [4–6]. In special psychiatric cases, such as atypical and/or pharmaco-resistant presentations, [^18^F]FDG-PET can also be useful for differential diagnosis with neurodegenerative diseases [7] or encephalitis [8, 9].

## Purpose

The present procedural guidelines summarize the current views of the EANM Neuro-Imaging Committee (NIC). The purpose of these guidelines is to assist nuclear medicine practitioners in making recommendations, performing, interpreting, and reporting results of [^18^F]FDG-PET imaging of the brain. The aim is to help achieve a high-quality standard of [^18^F]FDG brain imaging, to further increase the diagnostic impact of this technique in neurological, neurosurgical, and psychiatric practice.

The present document updated a previous version that has been published in 2009 [5]. These new guidelines develop advances in PET technology such as digital PET and hybrid PET/MR systems, advances in individual PET semiquantitative analysis, and current broadening clinical indications (e.g., for encephalitis and brain lymphoma). Further insight has also become available about hyperglycemia effects in patients who undergo brain [^18^F]FDG-PET. Accordingly, the patient preparation procedure has been updated. Finally, most typical brain patterns of metabolic changes are summarized for neurodegenerative diseases.

The present guidelines are specifically intended to present information related to the European practice. The information provided should be taken in the context of local conditions and regulations.

## Clinical indications

The following clinical indications especially integrate the EANM and European Academy of Neurology (EAN) recommendations for the use of brain [^18^F]FDG-PET [6].

### Common indications

Cognitive impairment and dementia. In neurodegenerative disorders such as Alzheimer’s disease (AD), changes in synaptic activity occur early in the course of the disease, when macrostructural brain changes cannot yet be detected. In this context, [^18^F]FDG-PET is viewed as a marker of neurodegeneration and progression, and currently included — with hippocampal volume measured with MRI (N) — in the amyloid-ß (A), tau (T), and neurodegeneration (ATN) classification scheme [10, 11]. A recent study suggests that [^18^F]FDG-PET is an independent biomarker to predict AD conversion in patients with mild cognitive impairment (MCI) along with amyloid-β and tau, independently of hippocampal volume [12] and of amyloid PET status [13, 14]. In the diagnostic work-up of patients with suspected AD dementia, the use of [^18^F]FDG-PET is complementary to other biomarkers, such as amyloid PET, CSF Aβ_42_, CSF Aβ_42_/Aβ_40_ ratio, CSF phosphorylated tau, and MRI [15]. [^18^F]FDG-PET is recommended to support early diagnosis of AD in MCI [16], early diagnosis of dementia with Lewy bodies (DLB), and frontotemporal lobar degeneration (FTLD) [16, 17]. [^18^F]FDG-PET is also recommended to support the differential diagnosis between (i) AD and FTLD, (ii) AD and DLB, (iii) FTLD and DLB; (iv) AD and vascular dementia when clinical and MRI data are inconclusive; and (v) differential diagnosis within neurodegenerative parkinsonian syndromes associated with dementia [16, 18–20]. In the framework of dementia work-up, a recent consensus algorithm has been proposed on suitable indications of [^18^F]FDG-PET, especially emphasizing its great value as a first-line evaluation when a non-AD disorder is clinically suspected [15, 21]. Typical topographic patterns of hypometabolism associated with AD, FTLD, and DLB are summarized in Table 1. Patterns of hypometabolism tend to mirror clinical presentations, and might also help to support diagnostic work-up of atypical AD in the framework of posterior cortical atrophy [22–24] and primary progressive aphasia [25] (also reported in Table 1). Beyond the differential diagnosis with vascular dementia, [^18^F]FDG-PET can also be used to help distinguish between cognitive impairment of degenerative diseases from nondegenerative origin, such as in traumatic brain injury (in correlation to MRI using PET/MRI device or fusion [26]), idiopathic normal pressure hydrocephalus (showing striatal hypometabolism with preserved cortical metabolism) [27], or depression. For the latter, studies report mild to moderate cortical hypometabolism in [^18^F]FDG-PET involving the frontal, temporal, insular, and cingulate areas, especially including the limbic areas, as well as the basal ganglia, in relation with the clinical severity and the therapeutic response [28–30]. Interestingly, a preserved [^18^F]FDG-PET scan has relevant negative predictive value, with less than 10% of patients progressing to degenerative dementia over 3 years [31]. For sensitivity, specificity, positive and negative predictive value of [^18^F]FDG-PET in this framework, the reader is referred to the EANM/EAN recommendations and to the original publications [6].

**Table 1:** Regions displaying typical hypometabolism in dementing disorders

Brain region	AD^1^	FTD	DLB	PCA	av-PPA	lv-PPA^6^	sv-PPA
Frontal lobe	*	✓	***		✓	✓	
Anterior cingulate gyrus		✓					
Temporoparietal cortex	✓		✓	✓	✓^5^		
Temporal lobe		✓	***			✓	✓
Posterior cingulate gyrus	✓	possible^8^	Island sign^2^	✓^3^		✓	
Precuneus	✓		✓				
Visual cortex			✓				
Occipital lobe	**		✓^7^	✓^4^			

AD, Alzheimer’s disease; FTD, frontotemporal dementia (behavioral variant); DLB, dementia with Lewy bodies; PCA, posterior cortical atrophy; PPA, primary progressive aphasia (av, agrammatic variant; lv, logopenic variant; sv, semantic variant)

^1^In heterogenous groups of AD patients, different patterns of hypometabolism have been observed [164]:

– AD-language dominant: left inferior frontal and left temporoparietal

– AD-visuospatial dominant: bilateral occipito-parieto-temporal, right posterior cingulate cortex/precuneus and right lateral parietal

*Frontal hypometabolism is present in more advanced stages of AD or in the frontal variant of AD (dorsolateral and orbitofrontal cortex) [164]

**Hypometabolism in the occipital lobe is present also in the posterior variant of AD (associative and primary visual cortex)

***DLB and PCA might show similar patterns of hypometabolism. However, hypometabolism in the orbitofrontal cortex and in the temporal pole is more frequent in DLB as compared with PCA [23]

^2^Posterior cingulate island sign refers to a relatively preserved metabolism in the posterior cingulate gyrus compared with the precuneus, a finding specific for DLB in comparison to AD [6, 17]. Beyond [^18^F]FDG-PET, the differential diagnosis between AD and DLB is also supported by dopamine transporter (DAT) imaging [165]. For the methods and indications, the reader is referred to the EANM/SNMMI guidelines for dopaminergic imaging in parkinsonian syndromes [166]

^3^In PCA, the cingulate island signs is also present, but it is often more asymmetric than in DLB [23]

^4^Hypometabolism of the visual association cortex is a typical finding in PCA [167]. In addition, asymmetry in the hypometabolism in the occipital cortex, as well as in the parietal cortex, is larger in PCA than in DLB [23]

^5^In non-fluent PPA/agrammatic variant of the primary progressive aphasia (av-PPA), the evidence of hypometabolism in the temporoparietal cortex is specific for the presence of AD pathology. A normal hypometabolism in the temporoparietal cortex is specific for the absence of AD pathology [168]

^6^In lvPPA, hypometabolism has been reported in the right medial temporal and posterior cingulate gyri, the left inferior, middle and superior temporal lobes, and left supramarginal gyrus [169]. Hypometabolic changes have been found to be more lateralized to the left hemisphere in amyloid PET-negative patients and more bilateral in amyloid PET-positive patients [170]

^7^Occipital hypometabolism in DLB is associated with visual hallucinations [171]

^8^Hypometabolism of posterior cingulate cortex can occur in FTD [172]

Movement disorders and parkinsonian syndromes. [^18^F]FDG-PET can be used for the differentiation between Parkinson’s disease (PD) and atypical parkinsonian syndromes such as multiple system atrophy (MSA), progressive supranuclear palsy (PSP), corticobasal syndrome (CBS), and the already mentioned DLB [32]. Typical topographic patterns of cortical and subcortical changes in glucose metabolism of parkinsonian conditions are presented in Table 2, including PD. These patterns have been described based on qualitative interpretation and semiquantitative analysis using voxel-based analysis or principal component analyses with the estimation of spatial covariance patterns (i.e. metabolic connectivity) [33–40]. Cortical hypometabolism in temporo-occipital and parietal regions has also been described in PD-MCI or PD patients that develop dementia at follow-up [33].

**Table.2:** Typical glucose metabolism patterns in parkinsonian disorders

	PD	PSP	MSA	CBS
Striatum	↑^1^	↓^2^	↓^3^	↓^4^
Thalamus	↑	↓		
Midbrain		↓		
Frontal lobe		↓		↓*
Medial frontal cortex		↓		
Sensorimotor cortex	↑**			↓*
Parietal cortex				↓*
Cerebellum	↑**		↓^5^	***

*Metabolic pattern of DLB is reported in* Table 1

^1^In PD the relative increase in metabolism is observed in the globus pallidus/putamen. The biological correlate of the relative increase in metabolism is an increase in striato-pallidal signaling due to loss of nigrostriatal input

^2^In PSP, the hypometabolism is observed in the caudate

^3^In MSA, the putamen is the striatal region typically affected in the parkinsonian variant of MSA (MSA-P)

^4^In CBS, the hypometabolism involves the whole striatum in the most typical presentation and is asymmetric, as well as the * involved hypometabolic cortex

^5^The hypometabolism in the cerebellum is more typically observed in the cerebellar form of MSA (MSA-C)

**A relative increase of metabolism in the sensorimotor cortex and cerebellum is associated more specifically to tremor

***Crossed cerebellar diaschisis can be observed in more advanced cases with severe cortical and subcortical hypometabolism

Other neurodegenerative motor diseases. The clinical use of [^18^F]FDG-PET as biomarker is still in its infancy in other neurodegenerative diseases, such as amyotrophic lateral sclerosis (ALS) and Huntington’s disease (HD) [41]. In ALS, the brain metabolic pattern consists of hypometabolism in the primary, pre- and supplementary motor cortices extending to the frontoparietal cortex, and hypermetabolism in the medial temporal cortex, cerebellum, and brainstem. There is a continuum between ALS and frontotemporal dementia in which 50% of patients have minor cognitive and behavioral changes, while 10–15% have overt frontotemporal dementia [42]. In the clinical setting, [^18^F]FDG brain PET has a prognostic value as patients with frontotemporal hypometabolism have a worse prognosis due to associated frontotemporal dementia. Although the metabolic pattern is able to discriminate patients with from controls with an accuracy higher than 90% [43–45], the diagnostic value is limited as the brain metabolic pattern is similar between diseases that mimic the symptoms of ALS (ALS-mimics) and ALS patients [46]. In HD, [^18^F]FDG-PET has only limited clinical value; however, HD patients with atypical (i.e., behavioral/psychiatric) presentations might have [^18^F]FDG-PET in the suspicion of other diseases, and the nuclear medicine physician should be able to recognize a peculiar pattern of hypometabolism associated with HD. It has been described that the striatal hypometabolism present in HD may identify carriers who will develop HD [47–51]. Besides striatal hypometabolism, HD is associated with a decreased cortical metabolism and an increased thalamic, occipital, and cerebellar metabolism [48]. The respective hypo- and hypermetabolism gradually increases during disease progression, and aid the selection of patients for clinical trials [52].

Epilepsy. The common indication is the presurgical evaluation of focal pharmaco-resistant epilepsy in adults and children to identify the epileptogenic zone using interictal injection [4, 53–56]. With a better spatial resolution, [^18^F]FDG-PET has also higher sensitivity than interictal perfusion SPECT, especially in temporal lobe epilepsy (TLE) (84 vs. 66% in a meta-analysis study) [57]. Uncoupling of blood flow and metabolism is moreover suspected in epilepsy, with more pronounced cerebral reduction in glucose metabolism than in perfusion. Of note, the interictal brain PET hypometabolism corresponds with the entire irritative zone (i.e., the epileptogenic zone and subsequent neural networks involved in the generation of interictal paroxysms). In this line, the extension of hypometabolism to areas beyond the temporal lobe is often found in patients with focal epilepsy but nevertheless with a great correlation to clinical presentations and stereo-EEG [53, 54]. Performance is lower in extra-TLE with identification of the epileptogenic zone in 38–67% of cases [4]. [^18^F]FDG-PET is of particular interest in suspected focal cortical dysplasia, also in children, and especially in case of (apparent) negative MRI [54]. Correlation to brain MRI using PET/MR device or image fusion techniques is particularly important in this framework to identify initially unknown lesions. Interestingly, the clinical outcome of cases with positive PET and negative MRI is similar to those with positive MRI [58, 59]. Finally, [^18^F]FDG-PET has good prognostic value for postsurgical outcome, especially in case of limited hypometabolism extent [53, 60–62], and also provides a prognostic value on cognition with more limited hypometabolism associated with a better postoperative cognitive status [63, 64].

Encephalitis, including autoimmune encephalitis (AE), and infectious and postinfectious encephalitis, as well frontier presentations (e.g., Morvan syndrome [65]) and differential diagnosis of inflammatory encephalopathies (e.g., neuro-lupus [66]; see Appendix of [67] for the whole spectrum of these disorders). [^18^F]FDG-PET is especially relevant in patients with negative or inconclusive MRI [68]. A recent systematic review and meta-analysis confirms a sensitivity of 80–90% of [^18^F]FDG-PET with a typical pattern associating global hypometabolism to striatal and limbic relative hypermetabolism [9], with also a specificity of 82% against MCI [69]. Medial temporal changes have been preferentially associated with autoantibodies against intracellular antigens [70]. This metabolic profile is also used in the follow-up to evaluate therapeutic efficacy, while whole-body PET is performed to identify cancer in paraneoplastic syndromes or systemic inflammatory/infectious localizations [71].

Neuro-oncology*.* Due to the high physiological uptake of [^18^F]FDG in normal brain gray matter and variable uptake by inflammatory lesions, [^18^F]FDG-PET has a more limited impact than amino-acid PET — when available — in the imaging of gliomas [72]. Better contrast between tumor and normal brain tissue as well between gray and white matter can be obtained with a longer time interval from FDG administration to data acquisition (e.g., 60 min up to several hours for tumors) [73]. A standardized acquisition protocol is nevertheless recommended with a fixed time for starting the acquisition to improve the comparability from different patients or repeated scans. Dedicated procedural guidelines have discussed the role and limitation of [^18^F]FDG-PET in patients with gliomas [74]. If amino-acid PET is not available, [^18^F]FDG-PET can be used at diagnosis, with increasing [^18^F]FDG uptake correlated to higher tumor grade and poorer prognosis [75], despite an overlap between grade I/II and grade III/IV gliomas, with also a prognostic value on survival at recurrence [76]. [^18^F]FDG-PET may be used to distinguish radiation necrosis from a recurrent tumor, with moderate additional value in comparison to MRI, usually at least 6 to 8 weeks after radiation therapy, with a pooled sensitivity and specificity of 84% on a recent meta-analysis [72, 74, 77, 78]. [^18^F]FDG-PET is also used at diagnosis to distinguish glioma from lymphoma, since most primary central nervous system lymphoma (PCNSL) lesions are highly [^18^F]FDG avid, with homogeneous uptake, and also from nonmalignant lesions in patients with AIDS (and particularly toxoplasma infection) [79]. A recent meta-analysis demonstrates high diagnostic accuracy of pre-treatment brain [^18^F]FDG-PET in PCNSL with pooled sensitivity, specificity, positive predictive value, and negative predictive value higher than 84% [80]. PET also contributes to the evaluation of the whole-body extension of the lymphoma with less than 5% of false positives in another recent meta-analysis [81].

### Contraindications

In case of PET/MR, patient safety information concerning magnetic field should be carefully checked prior to MRI (including the presence of devices not compatible such as pacemakers, neurostimulators, cochlear implants, non-MRI–compatible metal implants, pumps; in case of doubt for ocular metal pieces, a low dose X-ray can be performed).

Pregnancy. For any diagnostic procedure in a woman patient known or suspected to be pregnant, a critical decision is necessary to assess whether the benefits weigh against the possible harm.

Breastfeeding. Women should interrupt breastfeeding for the first 24 h after PET tracer application.

Lack of cooperation, or inability to cooperate. Claustrophobia and most important obesity can also be an obvious limitation, especially for PET/MR.

## Procedure

### Patient preparation

Pre-arrival. Patients should fast for at least 4 to 6 h to obtain optimal cerebral [^18^F]FDG uptake.

#### Information relevant for the procedure

History of diseases, especially neurological and psychiatric disorders, and current neurological and psychiatric status including clinical test results. Surgery, radiation, trauma of the brain.

#### History of diabetes, fasting state

Patients’ ability to lie still for 15 min to 1 h. If sedation is required, it should be performed as late as possible. The intention should be to administer [^18^F]FDG at least 15 min before the sedation.

Information about recent structural imaging studies (CT, MRI), fluid or molecular imaging biomarkers (CSF, plasma, amyloid or tau PET imaging), blood biochemistry indicative of metabolic dysfunction or systemic disease (e.g., hepatic, thyroid, renal), as well as about functional brain explorations (EEG, neuropsychometric tests) in specific conditions.

Ongoing necessary therapies are allowed, but current medications (and timing of their last administration) should be recorded. This information (including duration and dosage) is particularly relevant for sedatives, psychotropic pharmaceuticals, antiseizure medication, and corticosteroids. Possible effects of these medications on regional metabolic rate of glucose consumption (rCMRglc) have been suggested [82–84].

The use and dosage of corticosteroids might be particularly relevant for emerging indications of brain [^18^F]FDG-PET such as AE and PCNSL. In whole-body [^18^F]FDG-PET, possible false-negative results are well-known for inflammatory and autoimmune diseases treated with corticosteroids [85, 86]. For brain [^18^F]FDG-PET, and whenever clinically possible, it is also advised to scan patients before starting (or in any case as soon as possible after initiating) steroid treatment in case of AE and PCNSL. Regarding discontinuation of abuse drugs, it should be noted that reports are also available about the effects of early abstinence on regional brain metabolism. For example, a shifts in cortical-subcortical metabolic balance has been reported during early abstinence from chronic methamphetamine abuse [87]. Similarly, it has been shown that acute alcohol administration may decrease brain glucose utilization that may persist through early sobriety in heavy drinkers [88]. Finally, in the case of parkinsonian patients, it should be recorded whether the examination is conducted in a clinically defined “off” state, as levodopa might reduce glucose metabolism regionally [89, 90].

#### Preinjection

Blood glucose levels should be checked prior to [^18^F]FDG administration. When hyperglycemia is present (>160 mg/dl; >8.9 nmol/L), there is an increased competition of elevated plasma glucose with [^18^F]FDG on carrier enzyme and, because it is usually associated with high intracellular glucose levels, also on hexokinase enzyme. As a general rule, there is a decrease in [^18^F]FDG influx rate constant (K_1_) quantitatively paralleling blood glucose concentrations, resulting in deterioration of image quality with increasing glucose concentrations [91, 92]. In the case of hyperglycemia, [^18^F]FDG uptake is expected to be reduced in the whole brain (with stochastic noise increased). Decreased contrast between white and gray matter uptake can be found, which might, at least in theory, impact diagnostic accuracy [93, 94]. In recent years, some lines of evidence have suggested that hyperglycemia might more prominently enhance hypometabolism in the posterior parieto-occipital cortex [95, 96]. These regions encompass the typical AD hypometabolic pattern. Therefore, concerns have been raised about the impact of hyperglycemia on the accuracy of PET in patients with suspected AD [97]. Very few studies directly addressed this issue, and, to date, a measurable effect on scan interpretation has not been proven [98]. In any case, an examination should be postponed until a proper euglycemic state is reached. Notably, acute correction of hyperglycemia with insulin usually does not substantially improve brain image quality, probably because the normalization of an increased intracellular glucose level lags behind the normalization of the plasma glucose level [99]. Quantitation of regional cerebral glucose metabolism with [^18^F]FDG-PET also requires steady-state situations which are not maintained during falling plasma glucose levels after administration of insulin. Best results for clinical brain [^18^F]FDG-PET imaging in diabetics can be obtained in a euglycemic condition during correct therapeutic management [93, 99, 100]. Alternatively, brain perfusion SPECT should be considered, especially using a new generation of solid detectors [101].

Interestingly, it has been suggested that hyperglycemia obtained by intravenous infusion of 10% glucose solution could enhance detectability in patients with brain tumors [102]. Procedural guidelines for PET imaging in glioma should be considered for further details [74].

Before the scanning procedure, patients should void their bladder for maximum comfort during the study and to reduce radiation dose. Advising the patient to drink water and void the bladder again after the scanning session is also recommended to minimize radiation exposure.

When static scans are acquired, patients should be positioned comfortably in a quiet, dimly lit room at least 15 min before [^18^F]FDG administration and during the uptake phase of [^18^F]FDG (at least 20 min). The cannula for i.v. administration should be in place at least 10 min before [^18^F]FDG administration. Patients should be instructed not to speak, read, listen to music/sounds or otherwise be active. Patients should be awake. Closing the eyes could decrease metabolism in the occipital cortex, a cortical region that might be relevant for specific clinical conditions (as in DLB, characterized by hypometabolism of the occipital cortex) [103]. In any case, a consistent procedure is required in each center to maintain comparability between exams (eyes open/closed), also with respect to the normal control database if semiquantitative analyses based on voxels or regions/volumes of interest is performed.

For presurgical evaluation of epilepsy, close monitoring of the patient is required, ideally using continuous EEG recording. Such monitoring should start before injection, as soon as the patient arrives in the department, in order to ensure that [^18^F]FDG is not administered in an ictal/postictal stage. Monitoring should be maintained until at least 20 min after the radiopharmaceutical administration. MRI images acquired in combination with PET or prior to it, as well as a well-documented history of seizures before imaging are of critical importance for adequate image interpretation.

### Precautions and conscious sedation

Continuous supervision of patients during the whole scanning procedure is required. This is particularly important for patients with epilepsy and those with cognitive impairment.

In patients with limited ability to cooperate (e.g., due to their cognitive/behavioral disorders) and in whom no contraindications against medical sedation exist, it may be useful to apply conscious sedation (e.g., by a short-acting benzodiazepine such as i.v. midazolam). Administration should take place at least 20 min after tracer injection, preferably starting only a few minutes before data acquisition. Sedation should be used with caution and rather be avoided if dynamic acquisitions are performed for quantification of rCMRglc, because of the effects of the sedative on glucose metabolism and thus also on brain uptake of [^18^F]FDG. Appropriate monitoring by pulse oximetry should be performed to prevent cardiopulmonary depression, and appropriate antidote/emergency backup should be foreseen. The dose of sedation should be reduced in elderly patients. National regulations in terms of the influence of medical sedation on fitness to drive need to be considered.

### Radiopharmaceutical

#### Radionuclide

Fluorine-18

#### Radiopharmaceutical

2-[^18^F]fluoro-2-deoxy-D-glucose ([^18^F]FDG)

#### Recommended activity

Adults

The previous guidelines mentioned 125–250 MBq (typically 150 MBq) when scans are performed in 3D mode [5]. However, for high-sensitivity digital systems (TOF < 400 ps) and/or large axial field of view (also called total body) systems, activity might be lowered, probably by a factor of 2 or more, but limited data are at present available to give clear recommendations.

Children

Activity administered in MBq = 14 × multiple (dosage card)

Administered activities to children may also be lower in the case of high sensitivity digital systems, and should never exceed those recommended in the EANM dosage card v.01.02.2014 [104].

### Radiation dosimetry

Infants have a greater relative brain mass (10%) than adults (2–3%), so the percentage uptake of injected [^18^F]FDG is higher. Although in newborn infants, sufficient image quality may be achieved with an injected dose as low as 10 MBq [105] (in part also based on lower tissue attenuation), the advocated minimal dose stated from the pediatric dose card for the EANM is followed in these guidelines.

Estimated radiation doses in adults and children are shown in Table 3.

**Table.3:** Radiation exposure related to [^18^F]FDG

	Organ receiving the largest radiation dose mGy/MBq	Effective dose mSv/MBq
Adults	0.13 bladder wall	0.019
Children (5 yrs)	0.34 bladder wall	0.056

Calculations based on ICRP 128 – Table C31

For CT, the effective dose depends on collimation and scan type (axial, helical) [106] but is usually lower than 0.3 mSv for a so-called low-dose CT and typically around 2 mSv or lower for a diagnostic high-quality CT.

#### Data acquisition

Instrumentation

PET/CT and PET/MR systems with GSO, LSO, or LYSO crystals acquire images in list mode and in 3D. In addition, more recent PET/CT and PET/MR systems show increased sensitivity because of an increased axial field of views, silicon photomultipliers (“digital PET”) and/or use of time-of-flight technology [107–112]. These properties can be beneficial in those cases where the injected radioactivity and/or the acquisition time need to be reduced (e.g., pediatric cases or patients with limited ability to cooperate).

#### Positioning of the patient

Careful positioning of the patient’s head is critical, especially for cameras with a limited axial field of view. The orbitomeatal line is often used for standardized positioning, and the patient’s head can be fixed in place. To prevent movement artifacts, the patient should be instructed to avoid any movements of the head.

#### Type of acquisition

Depending on the clinical question and type of equipment, [^18^F]FDG-PET imaging may include:

- Static acquisition: A single set of tomographic images is obtained after brain uptake. Alternatively, the static scan can be divided into several frames to perform post-hoc movement correction. The static image should usually start with a fixed time, generally between 30- and 60-min postinjection (p.i.) for diagnostic purposes, and either evaluated by means of visual assessment or semiquantitative and/or voxel-based analysis.

- Dynamic acquisition: Multiple sequential sets of tomographic images are acquired from the time of administration of [^18^F]FDG until 60 or 90 min, usually. This acquisition is especially used when absolute quantification of rCMRglc is required generally for research purposes (*see paragraph on absolute quantification*) but also possibly in clinical settings to correct patient’s movements and perform parametric analysis.

#### Attenuation correction

This correction is mandatory for [^18^F]FDG PET brain imaging and is currently performed using CT- or MR-based attenuation correction.

- CT-based attenuation correction. PET/CT systems use CT scan for attenuation correction. The advantage of CT scan is that X-ray detection is not impacted by emission photons. Consequently, a CT scan can be performed after the radiopharmaceutical injection. A CT scan can be done for diagnostic purposes, using regular tube current, or only for attenuation correction (i.e., a low-dose CT) with low tube current (typically 10–30 mAs). The latter has the advantage to significantly reduce the radiation exposure well below 0.5 mSv for most systems. The choice of either type of CT scans depends on the purpose of imaging and the clinical indication. If anatomical information is already available, a low-dose CT for the purpose of attenuation correction can be considered. When performing PET/CT, it is recommended to check for movement between CT and the PET sessions, to avoid artifacts in the attenuation correction.

- MR-based attenuation correction [113]. For hybrid PET/MR systems, attenuation corrections (AC) need to be derived using either a dedicated MR sequence (MR-AC) or use of CT templates depending on the system’s available methods. In most of the systems, only MR-AC or CT templates are commercially available now, but improved and more advanced methods using special image reconstruction algorithms and artificial intelligence [114–118] are expected to be offered in future software updates. For MR-AC imaging, the body coil or a dedicated brain/head coil may be required. However, the best performing MR-AC methods work accurately for [^18^F]FDG brain PET imaging [119].

In all cases, it is strongly recommended to visually control the generated attenuation correction maps for unforeseen artifacts arising from metal or dental implants, missing air cavities, and missing bones — the latter is usually present in all MR-AC [120]. The reader should be aware of the possible qualitative and quantitative consequences of these artifacts and take them into consideration when reading or interpreting the PET images. For example, tracer uptake in cortical brain regions may be underestimated by about 20%, while uptake in the pons or cerebellum may show upward bias in case air cavities are not correctly considered by the MR-AC. The use of CT templates can overcome these limitations but may also be less accurate in case of abnormal bone anatomy, i.e., the templates assume healthy anatomical bone structures (absence of trauma). The latter can be overcome by separately making individual CT scans and inserting these into the PET/MR reconstruction pipeline for attenuation correction purposes. At present, such a procedure is not routinely available and formally approved on PET/MR systems. Pooling PET data collected on PET/CT and PET/MR systems (and on PET/MR systems with distinct methods for AC) should thus be performed with extreme caution as quantitative biases between these systems may be present.

#### Emission scan acquisition

As already noted, in the case of a static image acquisition procedure, the acquisition should not start earlier than 30 min p.i.. Better contrast between gray and white matter, as well between tumor and normal brain tissue, can be obtained with a longer time interval between [^18^F]FDG administration and data acquisition (e.g., 60 min up to several hours for tumors). A standardized acquisition protocol with a fixed time for acquisition start, generally between 30 min and 60 min p.i., is recommended to improve comparability between exams of different patients, follow-up scans, or different centers (e.g., as in multicenter trials). Standard protocols on modern hybrid PET/CT or PET/MR system includes list mode acquisition in 3D mode. The use of list mode acquisition is particularly useful in case of motion, allowing to exclude artifacts due to the patient’s movements. The duration of emission image acquisition should be related to the minimum required number of detected events. Typically, data are acquired over 10–15 min, possibly less if a higher [^18^F]FDG dose activity is administrated, for example, during an additional whole-body PET procedure. This whole-body PET/CT scan is particularly recommended in case of suspected paraneoplastic syndromes, lymphoma, or autoimmune/inflammatory/infectious systemic diseases also involving the brain (e.g., neurosarcoidosis). In special circumstances, when moderately agitated patients are examined, acquisition times down to 5 min can be used for diagnostic pattern evaluation [121].

In the case of a dynamic procedure, typically a 60-min 3D dynamic scan is acquired in list mode shortly before (10s) or simultaneously with the administration of [^18^F]FDG. During the PET acquisition, it is required to monitor head movement and to correct for any displacements. The use of a dedicated head holder or immobilization device to avoid or limit head movements is recommended. When available, a motion tracking system may be used to detect motion to retrospectively correct for any head displacements. It is beyond these guidelines to recommend motion correction methods as these are not widely available and/or generally accepted, as they are still under development. Yet, the reader should be aware that it is important to avoid and/or correct for patient motion in case of long dynamic PET brain studies. After completion of the dynamic scan, the list mode data can be binned (and reconstructed) into, e.g., 20 to 30 successive time frames to capture kinetics in brain tissue over time. Typically, time frames are short (~5 to 30s) on the first 5 min of the scan if input function is required, progressively increasing to about 300 s time frames at later uptake times.

#### Image reconstruction

Preferably, images are reconstructed using (ordered subset) iterative reconstruction including the use of TOF information, when available, and with an image matrix size of at least 128 × 128 pixels. Voxel size should be smaller than 2 mm in any direction at the smallest possible slice thickness. The number of iterations and subsets applied should guarantee sufficient convergence of the reconstructed data. It depends on the specific PET system and TOF performance. Resolution modeling is also part as a new feature in reconstruction software on many available PET/CT and PET/MR systems. This resolution modeling may be applied to enhance the detection of small abnormalities, but it is not yet recommended for quantitative evaluation due to pronounced Gibbs artifacts. At the time of writing these guidelines, dedicated brain PET harmonization is being explored and more specific recommendations may be provided soon by EARL [122]. Depending on PET system, a final image resolution of 4–6 mm FWHM (full width at half maximum) typically yields images of adequate resolution and noise. If movement artifacts are observed (especially in children or in patients with dementia or movement disorder), it can be helpful to reconstruct the data in short frames (e.g., 5-min frames) and evaluate only those frames that are not affected by patient motion or spatially align the individual frames prior to further analysis. In the case of severe patient motion, the attenuation correction (spatial mismatch between CT or MR-AC and PET) may no longer be accurate enough and cause image artifacts. It should be noted that non-attenuated series (which should be reconstructed and archived) could be useful in this setting to check for artifacts and for reporting.

#### Interventions

Usually, interventions are not necessary to answer routine clinical questions; they are mostly used in research. In the localization of eloquent cortical areas before surgery, stimulation paradigms like language or motor tasks can be performed. Currently, such activation imaging is mostly performed with functional MRI (fMRI). If performed with [^18^F]FDG-PET to image special clinical states [123–125], these paradigms usually start at the time of injection and have to be maintained for a time period of at least 20 min [126, 127].

### Image processing

#### Quantification procedures

The quantitative assessment of cerebral [^18^F]FDG/glucose metabolism requires, besides a dynamic emission scan, an arterial input function, i.e., the measurement of plasma [^18^F]FDG (over time) and glucose concentrations. There is a need for a calibration factor between scanner events in terms of detected events/voxel/s and in vitro (or online) measurements of plasma activity concentrations in counts/mL/s [128].

Glucose metabolism may be derived with either a Patlak plot or a pharmacokinetic model using the dynamic PET series and the arterial input function. The primary outcome parameter, the net influx rate constant Ki, then needs to be multiplied with plasma glucose levels to derive the rCMRglc which also takes into account the lumped constant. It is required to measure blood glucose levels during the scan or directly prior to or after the dynamic PET examination.

Although dynamic image acquisition from the start of injection up to usually 60 min p.i. is considered to be the most accurate procedure, most centers use simplified protocols based on static images in the clinical setting [129–131].

For brain tumor imaging, typically semiquantitative estimates of glucose metabolism like SUV (standardized uptake value) or SUVr (SUV relative to a normal brain region; usually in oncology to the contralateral metabolic uptake) are used. For such quantification, standardized acquisition times are required. The total activity of administered [^18^F]FDG and the patient’s height and weight for the estimation of body surface are also required. A calibration factor can also be applied as well as for comparative studies between different PET cameras. A static image is sufficient, acquired between 30 and 60 min p.i., typically. These semiquantitative estimates can be corrected for blood glucose concentration.

##### Absolute quantification

As already noted, estimation of the rCMRglc can be performed by compartmental modeling or using graphical analytic approaches. The quantification can be performed at both region of interest (ROI) and voxel level. In the ROI-based approach, rCMRglc is estimated in different brain regions by fitting the time-activity curve data using the measured arterial curve as input function. In the voxel-based approach, parametric images of rCMRglc can be calculated using Patlak analysis, graphical approach, or a basis function approach.

Lumped constant. A correction factor, the so-called lumped constant (LC) [132], can be used to convert [^18^F]FDG “metabolism” values to values reflecting glucose metabolism [130, 133]. The lumped constant might vary in pathological conditions. For instance, in malignant glioma, a higher LC than the one measured in the normal brain has been reported [134]. Under special physiological conditions, such as prolonged or extreme fasting, a reduction of glucose metabolism has been observed [135]. For instance, in one PET study conducted in obese patients before and after 3 weeks of fasting, a 50% decrease of rCMRglc was reported, with a 25% decrease of the LC [136].

Partial volume effect correction (PVC) can at best be applied in case of concomitant or prior acquisition of 3D T1-weighted MR scans. However, at present there is no consensus on which PVC method should be used or recommended (*see below*). Various methods exist with specific performances. It is also of utmost importance that MR data used during the PET analysis pipeline, either for the volume of interest definitions or PVC, are of sufficient quality and acquired with 1 × 1 × 1 mm voxels (or better). It is recommended to correct partial volume effect by the same MR scan and the same sequence to maintain reproducibility between exams. Of note, MR-free PVC corrections methods are available and can be considered as well, when MR data are not available or when harmonized MR image quality cannot be achieved [122].

#### Data display for analysis

Color scales typically used for the display of the images are gray scales (specially to detect lesion), or rainbow scales, with a continuous progression of the colors from low to high uptake.

A standardized image display, also in terms of upper/lower color scale thresholding, is advocated to ensure appropriate and best interpretable representation of the reconstructed dataset.

Internal landmarks can be used for reorientation to achieve a standardized image display. Reorientation procedures based on the inter-commissural line are generally used [137]. A proper reorientation of the coronal view is crucial for visual inspection of the scan as the presence of asymmetry between homologs structures in the two hemispheres is one of the cornerstones of visual reading. For a more accurate inspection of the medial temporal lobe, a second reorientation can be made along the hippocampal axis (the so-called Ohnishi reorientation in which a patients’ brain is reoriented 30 degrees upward with respect to the bi-commissural line on sagittal view [138]), for example, for temporal epilepsy or AD.

The display of additional coronal and sagittal images is recommended.

Three-dimensional display of the dataset can be helpful for more accurate topographic orientation in some clinical questions.

### Interpretation/semiquantification

#### Visual interpretation

The images should be critically examined during interpretation for the presence of movement or attenuation artifacts.

It is suitable to have a normal database available, preferably studied on the same type of camera, under the same acquisition circumstances (e.g., eyes open/closed) and using the same type of reconstruction and attenuation correction. Matching spatial resolution is the most important parameter needed for optimal database use. This allows assessment of normal variability of regional [^18^F]FDG uptake and improves diagnostic accuracy.

Variation in color scale, background subtraction, or change in contrast can be used to facilitate data interpretation. Data interpretation should take into consideration global changes and regional decreases or increases in [^18^F]FDG uptake. Tables 1 and 2 describe the most typical metabolic pattern in neurodegenerative diseases. Increased uptake can be observed in active epileptogenic foci, tumors, inflammation/infection, and physiologically activated brain areas.

Known morphological changes like atrophy should be considered for the full interpretation of the data. It is helpful to fuse [^18^F]FDG images with a MRI (or CT) scan of the individual performed within 6 months prior to the PET scan. In PET/CT and PET/MRI systems, fused PET/CT or PET/MRI images can be immediately visualized after image reconstruction without the need for specific software for image registration. Examples where image fusion is required are:

Presence of localized abnormalities with hypometabolism or hypermetabolism that can be related to, e.g., neuroinflammation, structural damage, atrophy, or infarcts.

Accurate evaluation of brain tumors and identification of the metabolically most active part of a brain tumor prior to biopsy.

Matching of cortical hypometabolism with morphological abnormalities on MRI or with the EEG focus for the planning of epilepsy surgery.

Localization of eloquent cortical areas (e.g., Broca’s area) prior to tumor resection.

##### Semiquantification and automated analysis

Tools for automated assessment and semiquantification can be used in the clinical settings to improve diagnostic performance especially of moderately skilled readers [16]. Several studies have investigated the added value of these tools in the clinical setting and showed higher specificity compared with visual reading, especially (but not only) for the identification of AD-related patterns, thereby increasing diagnostic confidence [139–143]. On the other hand, sensitivity of visual and automatic analyses has been shown to be relatively similar although visual analysis obviously is affected by the experience of the reader [16]. Indeed training and experience in the clinical settings are needed to report brain [^18^F]FDG PET especially given the possibility of subtle defects, as they sometimes occur in the earliest stages of neurodegenerative disease [16]. Supporting visual analysis with automated observer-independent approaches is especially suggested for less-skilled readers and, more in general, with the aim to reduce inter-reader variability [144].

When automated assessment of [^18^F]FDG-PET images is performed, several aspects should be taken into consideration:

- Semiquantitative/voxel-based approaches to [^18^F]FDG-PET analysis should always be used in conjunction with visual reading (considering visual reading as the first step for images evaluation and mandatory for quality control). Freeware and commercial software are available allowing for semiquantification or voxel-based analysis based on different methods [18, 145–148]. Available freeware software for voxel-based analyses often are non-CE licensed.
- Software for automated reading of [^18^F]FDG-PET is based on various approaches and has limitations or might introduce complexity (which might also represent a drawback when these tools aim at supporting less experienced readers). Automated systems can introduce artifacts (during post-processing or statistical analyses), thereby potentially generating erroneous results. It should be noted that very few studies have performed a head-to-head comparison between different software packages and therefore the main recommendation is, at present, to support visual reading through the use of an automated tool well-known by the user [16, 149].
- Tools for semiquantification and voxel analysis provide individual statistical maps (parametric or Z-score maps) aimed to support visual reading and to improve anatomical localization of regions of abnormal metabolism (more often hypometabolism). The user should consider that outputs and parametric maps generated by this software still need to be interpreted based on the hypo (or hyper)-metabolic patterns that were initially searched for also on native images. Readers should be aware that several software packages only report a decrease in metabolism and not an increase.
- Semiquantification of the brain [^18^F]FDG-PET either relying on a ROI-based or voxel-wise statistical evaluation generally require comparison between an individual patient’s PET image and age-matched databases of PET images obtained from healthy subjects. Commercial packages incorporate their own fixed healthy subject database although in some cases, only limited details are available about the control group. If the software used does not include an embedded normal controls database, the control group must be built locally in each center. This might be challenging and result in suboptimal control groups also from a clinical point of view (lack of follow-up of the controls; simple inclusion of normal scans rather than scans of healthy controls; controls recruited among patients who undergo [^18^F]FDG-PET for other indications). Finally, in recent years large databases of normal controls have been publicly shared in the framework of research projects and initiatives [150, 151]. The availability of these databases might contribute to a further spread of the use of voxel-based analyses also in a clinical setting; however, preparation, acquisition, and reconstruction parameters should be harmonized as much as possible with all parameters used to acquire the normal subject database to reduce the risk of generating bias and inconsistencies.
- Given the spatial resolution of PET and the size of the brain structures that need to be inspected, the partial volume effect may degrade “quantitative” accuracy of PET images [152, 153]. Because of partial volume/spillover effect, the intensity of a particular voxel not only reflects the tracer concentration of that voxel but also that of the surrounding area. However, only some software packages for automated analysis of [^18^F]FDG PET include PVC utilities designed to correct for spillover effects caused by the limited spatial resolution of PET images. Partial volume effect is a potential confounding factor in PET imaging studies focused on neurodegenerative diseases, since it might become unclear whether any observed decrease in the image signal is caused by functional changes or atrophy. This potential confounding effect should be taken into consideration for the final interpretation of the scan, as atrophy may also be the result of other (non-neurodegenerative) pathophysiological mechanisms (aging, chronic ischemia, postencephalitis brain damage, etc.).
- Intensity normalization (or scaling) is needed to overcome the effect of biological and technical non-disease–related factors that can affect regional [^18^F]FDG concentrations [154]. Accordingly, scaling can be performed by normalizing to the whole brain (proportional or global mean scaling), to predefined reference regions known to be spared in specific clinical settings (i.e., cerebellum, brain stem, pons, primary sensorimotor cortex, or gray matter) or on a data-driven basis [155–159]. The underlying assumption is that the reference region used is unaffected by disease or method (e.g., MR-AC), which needs to be carefully assessed. Intensity normalization is particularly critical when evaluating patients who might show both regions of hypermetabolism and hypometabolism such as patients with AE [160]. In general, when using software for brain [^18^F]FDG PET semiquantification, it is mandatory to consider the count rate normalization approach used. Accordingly, each semiquantification output should be interpreted jointly with the visual reading result.
- Voxel-based analytical approaches based on freeware software, such as 3D-SSP (Neurostat®) and statistical parametric mapping (SPM), have been extensively used to determine abnormalities of regional [^18^F]FDG uptake in an observer-independent way and to improve diagnostic accuracy in several clinical settings [56, 146, 161]. Originally, Neurostat was developed as a stand-alone tool. Since version 8, SPM is also available as a stand-alone tool (it could previously run only within the Matlab environment). Although a detailed discussion of Neurostat and SPM is outside the scope of the present procedural GLs, given their wide use, some issues related to their peculiar features are given here:
  * 3D-SSP (Neurostat®) provides a stereotactic surface projection displays. This tool was specifically designed for single-subject analysis and includes a group of controls (as well as the possibility to replace this build-in group with a database of local controls). It displays results using different reference regions for normalization (whole brain, pons, thalami, and cerebellum) allowing the user to appreciate the effect of the different reference regions on the final results (*see above*).
  * SPM was originally designed for voxel-based group comparisons. However, in more recent years, several studies have validated its use for single-subject analysis which can be implemented to support visual reading [4, 5]. A dementia-customized [^18^F]FDG-PET template has been made available in recent years including a balanced proportion of [^18^F]FDG-PET images from control subjects and patients [6]. The lack of a normal control group embedded within the tool is a potential limitation for its use in a clinical setting (*see above*). Finally, there is still a lack of standardization for SPM processing steps even though these steps can introduce bias and, more generally, can affect final results (this issue is particularly relevant when choosing a reference region instead of using global count density for intensity normalization; *see above*).

### Reporting

#### General

The report should include all pertinent information, including the name of the patient and other identifiers, such as date of birth, name of the referring physician(s), potentially interfering medications or abnormal glycemia, type of examination, date of examination, PET system used, administered activity, and patient history, including reasons for requesting the study.

#### Body of the report

Procedures and materials: Include in the report a description of image acquisition and processing, i.e., whether the images were acquired using a PET/CT or PET/MR system and procedure performed, such as arterial blood sampling. If CT is acquired for diagnostic purposes, include also a description of the scanning parameters including dosimetry. In the case of PET/MR, report the type of sequences that were acquired during the imaging session (e.g., structural MR, T1-w, T2-w, FLAIR, diffusion-weighted, arterial spin labeling, resting-state fMRI). If sedation is performed, briefly describe the procedure, including the type of medication and time of sedation in relation to the radiotracer injection. In epileptic patients, briefly describe the procedure of EEG recording, when performed.

Findings: Describe whether [^18^F]FDG-PET findings are normal or abnormal. If findings are abnormal, describe the location and intensity of abnormal [^18^F]FDG uptake. Functional topography can be used as well as anatomical descriptions. State quantitative or semiquantitative measures if performed. If the CT is acquired for diagnostic purposes, it is recommended to also include a description of the findings. In case of PET/MR, a description of the findings evaluated on structural MR images should be included.

Limitations: Where appropriate, identify factors that can limit the sensitivity and specificity of the examination (i.e., movement, small lesion size, systemic disease but also the deviation from standard procedure).

Clinical issues: The report should address or answer any pertinent clinical issues raised in the request for the imaging examination.

Comparative data: Comparisons with previous examinations and reports, if available, have to be part of the report. Furthermore, results of morphological imaging modalities should also be considered for interpretation. Nondiagnostic CT scans only used for attenuation in PET/CT should be used with caution for structural interpretation.

Interpretation and conclusion: If the PET examination presents a generally accepted disease pattern, this should be said in the conclusion and, if possible, using a statement that indicates the most probable diagnosis considering the pattern only in the context of the clinical presentation and hypothesis. Any (subjective) interpretation not based on such criteria has to be explicitly stated and considered as hypothetical. A differential diagnosis should be given when appropriate. When appropriate, follow-up or additional studies should be recommended to clarify or confirm the suspected diagnosis.

### Quality control

Quality control is described in previous guidelines [162].

Sources of error [163]:

- Unintended cerebral activation (i.e., visual or motor activation)
- Artifacts (patient movement during PET acquisition or between PET and CT/MRI, camera-related, induced by inappropriate processing) as well as MR-based AC biases (missing bones, air cavities, metal implants, etc.)
- Psychotropic drugs or corticosteroid use
- Sedation
- Incomplete intravenous tracer injection
- No or insufficient attenuation correction
- Soft tissue or skull uptake following surgery in the area of the skull or brain
- Recent radio- or chemotherapy

## References

[1] Zimmer ER, Parent MJ, Souza DG, Leuzy A, Lecrux C, Kim HI (2017). [(18)F]FDG PET signal is driven by astroglial glutamate transport. Nat Neurosci.

[2] Sweeney MD, Montagne A, Sagare AP, Nation DA, Schneider LS, Chui HC (2019). Vascular dysfunction-the disregarded partner of Alzheimer's disease. Alzheimers Dement.

[3] Xiang X, Wind K, Wiedemann T, Blume T, Shi Y, Briel N, et al. Microglial activation states drive glucose uptake and FDG-PET alterations in neurodegenerative diseases. Sci Transl Med. 2021;13:eabe5640. 10.1126/scitranslmed.abe5640. 10.1126/scitranslmed.abe5640 34644146

[4] Verger A, Lagarde S, Maillard L, Bartolomei F, Guedj E (2018). Brain molecular imaging in pharmacoresistant focal epilepsy: current practice and perspectives. Rev Neurol.

[5] Varrone A, Asenbaum S, Vander Borght T, Booij J, Nobili F, Någren K (2009). EANM procedure guidelines for PET brain imaging using [18F]FDG, version 2. Eur J Nucl Med Mol Imaging.

[6] Nobili F, Arbizu J, Bouwman F, Drzezga A, Agosta F, Nestor P (2018). European association of nuclear medicine and european academy of neurology recommendations for the use of brain. Eur J Neurol.

[7] Ortner MM (1750). The use of (18)F-FDG PET in the diagnostic workup of Alzheimer's dementia. Methods Mol Biol.

[8] Endres D, Rauer S, Kern W, Venhoff N, Maier SJ, Runge K (2019). Psychiatric presentation of anti-NMDA receptor encephalitis. Front Neurol.

[9] Bordonne M, Chawki MB, Doyen M, Kas A, Guedj E, Tyvaert L, et al. Brain (18)F-FDG PET for the diagnosis of autoimmune encephalitis: a systematic review and a meta-analysis. Eur J Nucl Med Mol Imaging. 2021. 10.1007/s00259-021-05299-y. 10.1007/s00259-021-05299-y 33677643

[10] Jack CR, Bennett DA, Blennow K, Carrillo MC, Dunn B, Haeberlein SB (2018). NIA-AA research framework: toward a biological definition of Alzheimer's disease. Alzheimers Dement.

[11] Tan MS, Ji X, Li JQ, Xu W, Wang HF, Tan CC (2020). Longitudinal trajectories of Alzheimer's ATN biomarkers in elderly persons without dementia. Alzheimers Res Ther.

[12] Ou YN, Xu W, Li JQ, Guo Y, Cui M, Chen KL (2019). FDG-PET as an independent biomarker for Alzheimer's biological diagnosis: a longitudinal study. Alzheimers Res Ther.

[13] Caroli A, Prestia A, Galluzzi S, Ferrari C, van der Flier WM, Ossenkoppele R (2015). Mild cognitive impairment with suspected nonamyloid pathology (SNAP): prediction of progression. Neurology..

[14] Iaccarino L, Sala A, Perani D (2019). Initiative AsDN. Predicting long-term clinical stability in amyloid-positive subjects by FDG-PET. Ann Clin Transl Neurol.

[15] Chételat G, Arbizu J, Barthel H, Garibotto V, Law I, Morbelli S (2020). Amyloid-PET and (18)F-FDG-PET in the diagnostic investigation of Alzheimer's disease and other dementias. Lancet Neurol.

[16] Nobili F, Festari C, Altomare D, Agosta F, Orini S, Van Laere K (2018). Automated assessment of FDG-PET for differential diagnosis in patients with neurodegenerative disorders. Eur J Nucl Med Mol Imaging.

[17] Gjerum L, Frederiksen KS, Henriksen OM, Law I, Anderberg L, Andersen BB (2020). A visual rating scale for cingulate island sign on 18F-FDG-PET to differentiate dementia with Lewy bodies and Alzheimer's disease. J Neurol Sci.

[18] Herholz K, Salmon E, Perani D, Baron JC, Holthoff V, Frölich L (2002). Discrimination between Alzheimer dementia and controls by automated analysis of multicenter FDG PET. Neuroimage..

[19] Mosconi L, Tsui WH, Herholz K, Pupi A, Drzezga A, Lucignani G (2008). Multicenter standardized 18F-FDG PET diagnosis of mild cognitive impairment, Alzheimer's disease, and other dementias. J Nucl Med.

[20] Garibotto V, Herholz K, Boccardi M, Picco A, Varrone A, Nordberg A (2017). Clinical validity of brain fluorodeoxyglucose positron emission tomography as a biomarker for Alzheimer's disease in the context of a structured 5-phase development framework. Neurobiol Aging.

[21] Chételat G, Arbizu J, Barthel H, Garibotto V, Lammertsma AA, Law I (2021). Finding our way through the labyrinth of dementia biomarkers. Eur J Nucl Med Mol Imaging.

[22] Singh TD, Josephs KA, Machulda MM, Drubach DA, Apostolova LG, Lowe VJ (2015). Clinical, FDG and amyloid PET imaging in posterior cortical atrophy. J Neurol.

[23] Whitwell JL, Graff-Radford J, Singh TD, Drubach DA, Senjem ML, Spychalla AJ (2017). F-FDG PET in posterior cortical atrophy and dementia with lewy bodies. J Nucl Med.

[24] Migliaccio R, Agosta F, Basaia S, Cividini C, Habert MO, Kas A (2020). Functional brain connectome in posterior cortical atrophy. Neuroimage Clin..

[25] Bouwman F, Orini S, Gandolfo F, Altomare D, Festari C, Agosta F (2018). Diagnostic utility of FDG-PET in the differential diagnosis between different forms of primary progressive aphasia. Eur J Nucl Med Mol Imaging.

[26] Salat DH, Robinson ME, Miller DR, Clark DC, McGlinchey RE (2017). Neuroimaging of deployment-associated traumatic brain injury (TBI) with a focus on mild TBI (mTBI) since 2009. Brain Inj.

[27] Townley RA, Botha H, Graff-Radford J, Boeve BF, Petersen RC, Senjem ML (2018). F-FDG PET-CT pattern in idiopathic normal pressure hydrocephalus. Neuroimage Clin.

[28] Videbech P (2000). PET measurements of brain glucose metabolism and blood flow in major depressive disorder: a critical review. Acta Psychiatr Scand.

[29] Kimbrell TA, Ketter TA, George MS, Little JT, Benson BE, Willis MW (2002). Regional cerebral glucose utilization in patients with a range of severities of unipolar depression. Biol Psychiatry.

[30] Kennedy SH, Evans KR, Krüger S, Mayberg HS, Meyer JH, McCann S (2001). Changes in regional brain glucose metabolism measured with positron emission tomography after paroxetine treatment of major depression. Am J Psychiatry.

[31] Silverman DH, Small GW, Chang CY, Lu CS, Kung De Aburto MA (2001). Chen W, et al. positron emission tomography in evaluation of dementia: regional brain metabolism and long-term outcome. JAMA..

[32] Verger A, Grimaldi S, Ribeiro MJ, Frismand S, Guedj E. SPECT/PET molecular imaging for parkinsonism: a fast-developing field. Ann Neurol. 2021. 10.1002/ana.26187. 10.1002/ana.26187 PMC9291534 34338333

[33] Walker Z, Gandolfo F, Orini S, Garibotto V, Agosta F, Arbizu J (2018). Clinical utility of FDG PET in Parkinson's disease and atypical parkinsonism associated with dementia. Eur J Nucl Med Mol Imaging.

[34] Whitwell JL, Höglinger GU, Antonini A, Bordelon Y, Boxer AL, Colosimo C (2017). Radiological biomarkers for diagnosis in PSP: where are we and where do we need to be?. Mov Disord.

[35] Niethammer M, Eidelberg D (2012). Metabolic brain networks in translational neurology: concepts and applications. Ann Neurol.

[36] Ge J, Wu J, Peng S, Wu P, Wang J, Zhang H (2018). Reproducible network and regional topographies of abnormal glucose metabolism associated with progressive supranuclear palsy: multivariate and univariate analyses in American and Chinese patient cohorts. Hum Brain Mapp.

[37] Niethammer M, Tang CC, Feigin A, Allen PJ, Heinen L, Hellwig S (2014). A disease-specific metabolic brain network associated with corticobasal degeneration. Brain..

[38] Martí-Andrés G, van Bommel L, Meles SK, Riverol M, Valentí R, Kogan RV (2020). Multicenter validation of metabolic abnormalities related to psp according to the MDS-PSP criteria. Mov Disord.

[39] Eckert T, Barnes A, Dhawan V, Frucht S, Gordon MF, Feigin AS (2005). FDG PET in the differential diagnosis of parkinsonian disorders. Neuroimage..

[40] Eckert T, Tang C, Ma Y, Brown N, Lin T, Frucht S (2008). Abnormal metabolic networks in atypical parkinsonism. Mov Disord.

[41] Agosta F, Altomare D, Festari C, Orini S, Gandolfo F, Boccardi M (2018). Clinical utility of FDG-PET in amyotrophic lateral sclerosis and Huntington's disease. Eur J Nucl Med Mol Imaging.

[42] van Es MA, Hardiman O, Chio A, Al-Chalabi A, Pasterkamp RJ, Veldink JH (2017). Amyotrophic lateral sclerosis. Lancet..

[43] Pagani M, Oberg J, De Carli F, Calvo A, Moglia C, Canosa A (2016). Metabolic spatial connectivity in amyotrophic lateral sclerosis as revealed by independent component analysis. Hum Brain Mapp.

[44] Van Weehaeghe D, Ceccarini J, Delva A, Robberecht W, Van Damme P, Van Laere K (2016). Prospective validation of 18F-FDG brain PET discriminant analysis methods in the diagnosis of amyotrophic lateral sclerosis. J Nucl Med.

[45] Van Laere K, Vanhee A, Verschueren J, De Coster L, Driesen A, Dupont P (2014). Value of 18fluorodeoxyglucose-positron-emission tomography in amyotrophic lateral sclerosis: a prospective study. JAMA Neurol.

[46] Van Weehaeghe D, Devrome M, Schramm G, De Vocht J, Deckers W, Baete K (2020). Combined brain and spinal FDG PET allows differentiation between ALS and ALS mimics. Eur J Nucl Med Mol Imaging.

[47] Tang CC, Feigin A, Ma Y, Habeck C, Paulsen JS, Leenders KL (2013). Metabolic network as a progression biomarker of premanifest Huntington's disease. J Clin Invest.

[48] Feigin A, Tang C, Ma Y, Mattis P, Zgaljardic D, Guttman M (2007). Thalamic metabolism and symptom onset in preclinical Huntington's disease. Brain..

[49] Herben-Dekker M, van Oostrom JC, Roos RA, Jurgens CK, Witjes-Ané MN, Kremer HP (2014). Striatal metabolism and psychomotor speed as predictors of motor onset in Huntington's disease. J Neurol.

[50] Ciarmiello A, Giovacchini G, Orobello S, Bruselli L, Elifani F, Squitieri F (2012). 18F-FDG PET uptake in the pre-Huntington disease caudate affects the time-to-onset independently of CAG expansion size. Eur J Nucl Med Mol Imaging.

[51] Hellem MNN, Vinther-Jensen T, Anderberg L, Budtz-Jørgensen E, Hjermind LE, Larsen VA (2021). Hybrid 2-[18F] FDG PET/MRI in premanifest Huntington's disease gene-expansion carriers: the significance of partial volume correction. PLoS One.

[52] Peralta C, Biafore F, Depetris TS, Bastianello M (2019). Recent advancement and clinical implications of 18FDG-PET in Parkinson's disease, atypical Parkinsonisms, and other movement disorders. Curr Neurol Neurosci Rep.

[53] Guedj E, Bonini F, Gavaret M, Trébuchon A, Aubert S, Boucekine M (2015). 18FDG-PET in different subtypes of temporal lobe epilepsy: SEEG validation and predictive value. Epilepsia..

[54] Lagarde S, Boucekine M, McGonigal A, Carron R, Scavarda D, Trebuchon A (2020). Relationship between PET metabolism and SEEG epileptogenicity in focal lesional epilepsy. Eur J Nucl Med Mol Imaging.

[55] Henry TR, Votaw JR (2004). The role of positron emission tomography with [18F]fluorodeoxyglucose in the evaluation of the epilepsies. Neuroimaging Clin N Am.

[56] Van Paesschen W, Dupont P, Sunaert S, Goffin K, Van Laere K (2007). The use of SPECT and PET in routine clinical practice in epilepsy. Curr Opin Neurol.

[57] Spencer SS (1994). The relative contributions of MRI, SPECT, and PET imaging in epilepsy. Epilepsia..

[58] Gok B, Jallo G, Hayeri R, Wahl R, Aygun N (2013). The evaluation of FDG-PET imaging for epileptogenic focus localization in patients with MRI positive and MRI negative temporal lobe epilepsy. Neuroradiology..

[59] LoPinto-Khoury C, Sperling MR, Skidmore C, Nei M, Evans J, Sharan A (2012). Surgical outcome in PET-positive, MRI-negative patients with temporal lobe epilepsy. Epilepsia..

[60] Dupont S, Semah F, Clémenceau S, Adam C, Baulac M, Samson Y (2000). Accurate prediction of postoperative outcome in mesial temporal lobe epilepsy: a study using positron emission tomography with 18fluorodeoxyglucose. Arch Neurol.

[61] Vinton AB, Carne R, Hicks RJ, Desmond PM, Kilpatrick C, Kaye AH (2007). The extent of resection of FDG-PET hypometabolism relates to outcome of temporal lobectomy. Brain..

[62] Tomás J, Pittau F, Hammers A, Bouvard S, Picard F, Vargas MI (2019). The predictive value of hypometabolism in focal epilepsy: a prospective study in surgical candidates. Eur J Nucl Med Mol Imaging.

[63] Akanuma N, Reed LJ, Marsden PK, Jarosz J, Adachi N, Hallett WA (2009). Hemisphere-specific episodic memory networks in the human brain: a correlation study between intracarotid amobarbital test and [(18)F]FDG-PET. J Cogn Neurosci.

[64] Weintrob DL, Saling MM, Berkovic SF, Berlangieri SU, Reutens DC (2002). Verbal memory in left temporal lobe epilepsy: evidence for task-related localization. Ann Neurol.

[65] Benedetti L, Franciotta D, Zoccarato M, Beronio A, Godani M, Schirinzi E (2015). Post-therapy normalization of brain FDG-PET in Morvan's syndrome. J Neurol Sci.

[66] Mauro D, Barbagallo G, Angelo DS, Sannino P, Naty S, Bruno C (2018). Role of positron emission tomography for central nervous system involvement in systemic autoimmune diseases: status and perspectives. Curr Med Chem.

[67] Graus F, Titulaer MJ, Balu R, Benseler S, Bien CG, Cellucci T (2016). A clinical approach to diagnosis of autoimmune encephalitis. Lancet Neurol.

[68] Morbelli S, Arbizu J, Booij J, Chen MK, Chetelat G, Cross DJ, et al. The need of standardization and of large clinical studies in an emerging indication of [(18)F]FDG PET: the autoimmune encephalitis. Eur J Nucl Med Mol Imaging. Germany; 2017. p. 353–7. 10.1007/s00259-016-3589-9 27924371

[69] De Leiris N, Ruel B, Vervandier J, Boucraut J, Grimaldi S, Horowitz T, et al. Decrease in the cortex/striatum metabolic ratio on [18F]-FDG PET: a biomarker of autoimmune encephalitis. Eur J Nucl Med Mol Imaging. 2021. 10.1007/s00259-021-05507-9. 10.1007/s00259-021-05507-9 34462791

[70] Baumgartner A, Rauer S, Mader I, Meyer PT (2013). Cerebral FDG-PET and MRI findings in autoimmune limbic encephalitis: correlation with autoantibody types. J Neurol.

[71] Morbelli S, Djekidel M, Hesse S, Pagani M, Barthel H (2016). (EANM) NCotEAoNM, et al. role of (18)F-FDG-PET imaging in the diagnosis of autoimmune encephalitis. Lancet Neurol.

[72] Albert NL, Weller M, Suchorska B, Galldiks N, Soffietti R, Kim MM (2016). Response assessment in neuro-oncology working group and european association for neuro-oncology recommendations for the clinical use of PET imaging in gliomas. Neuro-Oncology.

[73] Spence AM, Muzi M, Mankoff DA, O'Sullivan SF, Link JM, Lewellen TK (2004). 18F-FDG PET of gliomas at delayed intervals: improved distinction between tumor and normal gray matter. J Nucl Med.

[74] Law I, Albert NL, Arbizu J, Boellaard R, Drzezga A, Galldiks N, et al. Joint EANM/EANO/RANO practice guidelines/SNMMI procedure standards for imaging of gliomas using PET with radiolabelled amino acids and [18 F]FDG: version 1.0. Eur J Nucl Med Mol Imaging. 2019;46:540–57. 10.1007/s00259-018-4207-9. 10.1007/s00259-018-4207-9 PMC6351513 30519867

[75] Colavolpe C, Metellus P, Mancini J, Barrie M, Béquet-Boucard C, Figarella-Branger D (2012). Independent prognostic value of pre-treatment 18-FDG-PET in high-grade gliomas. J Neuro-Oncol.

[76] Colavolpe C, Chinot O, Metellus P, Mancini J, Barrie M, Bequet-Boucard C (2012). FDG-PET predicts survival in recurrent high-grade gliomas treated with bevacizumab and irinotecan. Neuro-Oncology.

[77] Kaschten B, Stevenaert A, Sadzot B, Deprez M, Degueldre C, Del Fiore G (1998). Preoperative evaluation of 54 gliomas by PET with fluorine-18-fluorodeoxyglucose and/or carbon-11-methionine. J Nucl Med.

[78] de Zwart PL, van Dijken BRJ, Holtman GA, Stormezand GN, Dierckx RAJO, Jan van Laar P (2020). Diagnostic accuracy of pet tracers for the differentiation of tumor progression from treatment-related changes in high-grade glioma: a systematic review and metaanalysis. J Nucl Med.

[79] Hoffman JM, Waskin HA, Schifter T, Hanson MW, Gray L, Rosenfeld S (1993). FDG-PET in differentiating lymphoma from nonmalignant central nervous system lesions in patients with AIDS. J Nucl Med.

[80] Gupta T, Manjali JJ, Kannan S, Purandare N, Rangarajan V. Diagnostic performance of pretreatment 18F-fluorodeoxyglucose positron emission tomography with or without computed tomography in patients with primary central nervous system lymphoma: updated systematic review and diagnostic test accuracy meta-analyses. Clin Lymphoma Myeloma Leuk. 2021. 10.1016/j.clml.2021.03.011. 10.1016/j.clml.2021.03.011 33947632

[81] Gupta T, Manjali JJ, Kannan S, Purandare N, Rangarajan V (2020). Diagnostic yield of extensive systemic staging including whole-body 18f-fluoro-deoxy-glucose positron emission tomography with or without computed tomography in patients with primary central nervous system lymphoma: systematic review and meta-analysis. Clin Lymphoma Myeloma Leuk.

[82] Spanaki MV, Siegel H, Kopylev L, Fazilat S, Dean A, Liow K (1999). The effect of vigabatrin (gamma-vinyl GABA) on cerebral blood flow and metabolism. Neurology..

[83] Liu S, Wang Y, Xu K, Ping F, Li F, Wang R (2018). Voxel-based comparison of brain glucose metabolism between patients with Cushing's disease and healthy subjects. Neuroimage Clin..

[84] Phelps ME, Huang SC, Hoffman EJ, Selin C, Sokoloff L, Kuhl DE (1979). Tomographic measurement of local cerebral glucose metabolic rate in humans with (F-18)2-fluoro-2-deoxy-D-glucose: validation of method. Ann Neurol.

[85] Jamar F, Buscombe J, Chiti A, Christian PE, Delbeke D, Donohoe KJ (2013). EANM/SNMMI guideline for 18F-FDG use in inflammation and infection. J Nucl Med.

[86] Clifford AH, Murphy EM, Burrell SC, Bligh MP, MacDougall RF, Heathcote JG (2017). Positron emission tomography/computerized tomography in newly diagnosed patients with giant cell arteritis who are taking glucocorticoids. J Rheumatol.

[87] Berman SM, Voytek B, Mandelkern MA, Hassid BD, Isaacson A, Monterosso J (2008). Changes in cerebral glucose metabolism during early abstinence from chronic methamphetamine abuse. Mol Psychiatry.

[88] Volkow ND, Wang GJ, Shokri Kojori E, Fowler JS, Benveniste H, Tomasi D (2015). Alcohol decreases baseline brain glucose metabolism more in heavy drinkers than controls but has no effect on stimulation-induced metabolic increases. J Neurosci.

[89] Berding G, Odin P, Brooks DJ, Nikkhah G, Matthies C, Peschel T (2001). Resting regional cerebral glucose metabolism in advanced Parkinson's disease studied in the off and on conditions with [(18)F]FDG-PET. Mov Disord.

[90] Feigin A, Fukuda M, Dhawan V, Przedborski S, Jackson-Lewis V, Mentis MJ (2001). Metabolic correlates of levodopa response in Parkinson's disease. Neurology..

[91] Apostolova I, Lange C, Suppa P, Spies L, Klutmann S, Adam G (2018). Impact of plasma glucose level on the pattern of brain FDG uptake and the predictive power of FDG PET in mild cognitive impairment. Eur J Nucl Med Mol Imaging.

[92] Sarikaya I, Sarikaya A, Sharma P (2019). Assessing the effect of various blood glucose levels on. J Nucl Med Technol..

[93] Henriksen OM, Holm S, Marner L, Law I (2020). Effect of blood glucose and body weight on image quality in brain [18F]FDG PET imaging. Nucl Med Commun.

[94] Eskian M, Alavi A, Khorasanizadeh M, Viglianti BL, Jacobsson H, Barwick TD (2019). Effect of blood glucose level on standardized uptake value (SUV) in (18)F- FDG PET-scan: a systematic review and meta-analysis of 20,807 individual SUV measurements. Eur J Nucl Med Mol Imaging.

[95] Byun MS, Kim HJ, Yi D, Choi HJ, Baek H, Lee JH (2019). Region-specific association between basal blood insulin and cerebral glucose metabolism in older adults. Neuroimage Clin..

[96] Sarikaya I, Sarikaya A, Sharma P (2019). Assessing the effect of various blood glucose levels on (18)F-FDG activity in the brain, liver, and blood pool. J Nucl Med Technol.

[97] Biessels GJ, Nobili F, Teunissen CE, Simó R, Scheltens P (2020). Understanding multifactorial brain changes in type 2 diabetes: a biomarker perspective. Lancet Neurol.

[98] Viglianti BL, Wale DJ, Ma T, Johnson TD, Bohnen NI, Wong KK (2019). Effects of plasma glucose levels on regional cerebral 18F-fluorodeoxyglucose uptake: implications for dementia evaluation with brain PET imaging. Biomed Pharmacother.

[99] Cranston I, Marsden P, Matyka K, Evans M, Lomas J, Sonksen P (1998). Regional differences in cerebral blood flow and glucose utilization in diabetic man: the effect of insulin. J Cereb Blood Flow Metab.

[100] Hasselbalch SG, Knudsen GM, Videbaek C, Pinborg LH, Schmidt JF, Holm S (1999). No effect of insulin on glucose blood-brain barrier transport and cerebral metabolism in humans. Diabetes..

[101] Bordonne M, Chawki MB, Marie PY, Zaragori T, Roch V, Grignon R (2020). High-quality brain perfusion SPECT images may be achieved with a high-speed recording using 360° CZT camera. EJNMMI Phys..

[102] Ishizu K, Nishizawa S, Yonekura Y, Sadato N, Magata Y, Tamaki N (1994). Effects of hyperglycemia on FDG uptake in human brain and glioma. J Nucl Med.

[103] Hahn A, Gryglewski G, Nics L, Hienert M, Rischka L, Vraka C (2016). Quantification of task-specific glucose metabolism with constant infusion of 18F-FDG. J Nucl Med.

[104] Lassmann M, Treves ST, Group ESPDHW. Paediatric radiopharmaceutical administration: harmonization of the 2007 EANM paediatric dosage card (version 1.5.2008) and the 2010 North American consensus guidelines. Eur J Nucl Med Mol Imaging. 2014;41:1036–41. 10.1007/s00259-014-2731-9. 10.1007/s00259-014-2731-9 24599377

[105] Ruotsalainen U, Suhonen-Polvi H, Eronen E, Kinnala A, Bergman J, Haaparanta M (1996). Estimated radiation dose to the newborn in FDG-PET studies. J Nucl Med.

[106] Wu TH, Huang YH, Lee JJ, Wang SY, Wang SC, Su CT (2004). Radiation exposure during transmission measurements: comparison between CT- and germanium-based techniques with a current PET scanner. Eur J Nucl Med Mol Imaging.

[107] Rausch I, Ruiz A, Valverde-Pascual I, Cal-González J, Beyer T, Carrio I (2019). Performance evaluation of the vereos PET/CT system according to the NEMA NU2-2012 standard. J Nucl Med.

[108] Hsu DFC, Ilan E, Peterson WT, Uribe J, Lubberink M, Levin CS (2017). Studies of a next-generation silicon-photomultiplier-based time-of-flight PET/CT system. J Nucl Med.

[109] van Sluis J, de Jong J, Schaar J, Noordzij W, van Snick P, Dierckx R (2019). Performance characteristics of the digital biograph vision PET/CT system. J Nucl Med.

[110] van Sluis J, Boellaard R, Somasundaram A, van Snick PH, Borra RJH, Dierckx RAJO (2020). Image quality and semiquantitative measurements on the biograph vision PET/CT system: initial experiences and comparison with the biograph mCT. J Nucl Med.

[111] Chen S, Hu P, Gu Y, Yu H, Shi H (2020). Performance characteristics of the digital uMI550 PET/CT system according to the NEMA NU2-2018 standard. EJNMMI Phys..

[112] Salvadori J, Imbert L, Perrin M, Karcher G, Lamiral Z, Marie PY (2019). Head-to-head comparison of image quality between brain. EJNMMI Res.

[113] Rausch I, Quick HH, Cal-Gonzalez J, Sattler B, Boellaard R, Beyer T (2017). Technical and instrumentational foundations of PET/MRI. Eur J Radiol.

[114] Chen Y, Ying C, Binkley MM, Juttukonda MR, Flores S, Laforest R (2021). Deep learning-based T1-enhanced selection of linear attenuation coefficients (DL-TESLA) for PET/MR attenuation correction in dementia neuroimaging. Magn Reson Med.

[115] Mecheter I, Alic L, Abbod M, Amira A, Ji J (2020). MR image-based attenuation correction of brain pet imaging: review of literature on machine learning approaches for segmentation. J Digit Imaging.

[116] Hofmann M, Pichler B, Schölkopf B, Beyer T (2009). Towards quantitative PET/MRI: a review of MR-based attenuation correction techniques. Eur J Nucl Med Mol Imaging.

[117] De Luca F, Bolin M, Blomqvist L, Wassberg C, Martin H, Falk DA (2020). Validation of PET/MRI attenuation correction methodology in the study of brain tumours. BMC Med Imaging.

[118] Ladefoged CN, Hansen AE, Henriksen OM, Bruun FJ, Eikenes L, Øen SK (2020). AI-driven attenuation correction for brain PET/MRI: clinical evaluation of a dementia cohort and importance of the training group size. Neuroimage..

[119] Ladefoged CN, Law I, Anazodo U, St Lawrence K, Izquierdo-Garcia D, Catana C (2017). A multi-Centre evaluation of eleven clinically feasible brain PET/MRI attenuation correction techniques using a large cohort of patients. Neuroimage..

[120] Ladefoged CN, Andersen FL, Kjær A, Højgaard L, Law I (2017). Resolute PET/MRI attenuation correction for O-(2-(18)F-fluoroethyl)-L-tyrosine (FET) in brain tumor patients with metal implants. Front Neurosci.

[121] Chen WP, Matsunari I, Noda A, Yanase D, Yajima K, Takeda N (2005). Rapid scanning protocol for brain (18)F-FDG PET: a validation study. J Nucl Med.

[122] Verwer EE, Golla SSV, Kaalep A, Lubberink M, van Velden FHP, Bettinardi V, et al. Harmonisation of PET/CT contrast recovery performance for brain studies. Eur J Nucl Med Mol Imaging. 2021. 10.1007/s00259-021-05201-w. 10.1007/s00259-021-05201-w PMC8263427 33517517

[123] Verger A, Guedj E (2018). The renaissance of functional F-18-FDG PET brain activation imaging. Eur J Nucl Med Mol Imaging.

[124] Rousseau PF, Malbos E, Verger A, Nicolas F, Lancon C, Khalfa S (2019). Increase of precuneus metabolism correlates with reduction of PTSD symptoms after EMDR therapy in military veterans: an 18F-FDG PET study during virtual reality exposure to war. Eur J Nucl Med Mol Imaging.

[125] Verger A, Malbos E, Reynaud E, Mallet P, Mestre D, Pergandi JM (2018). Brain metabolism and related connectivity in patients with acrophobia treated by virtual reality therapy: an (18)F-FDG PET pilot study sensitized by virtual exposure. EJNMMI Res.

[126] Schreckenberger M, Spetzger U, Sabri O, Meyer PT, Zeggel T, Zimny M (2001). Localisation of motor areas in brain tumour patients: a comparison of preoperative [18F]FDG-PET and intraoperative cortical electrostimulation. Eur J Nucl Med.

[127] Herholz K, Pietrzyk U, Karbe H, Würker M, Wienhard K, Heiss WD (1994). Individual metabolic anatomy of repeating words demonstrated by MRI-guided positron emission tomography. Neurosci Lett.

[128] Schelbert HR, Hoh CK, Royal HD, Brown M, Dahlbom MN, Dehdashti F (1998). Procedure guideline for tumor imaging using fluorine-18-FDG. Society of Nuclear Medicine. J Nucl Med.

[129] Phelps ME (2000). PET: the merging of biology and imaging into molecular imaging. J Nucl Med.

[130] Lucignani G, Schmidt KC, Moresco RM, Striano G, Colombo F, Sokoloff L (1993). Measurement of regional cerebral glucose utilization with fluorine-18-FDG and PET in heterogeneous tissues: theoretical considerations and practical procedure. J Nucl Med.

[131] Henry TR, Engel J, Mazziotta JC (1993). Clinical evaluation of interictal fluorine-18-fluorodeoxyglucose PET in partial epilepsy. J Nucl Med.

[132] Graham MM, Muzi M, Spence AM, O'Sullivan F, Lewellen TK, Link JM (2002). The FDG lumped constant in normal human brain. J Nucl Med.

[133] Hasselbalch SG, Madsen PL, Knudsen GM, Holm S, Paulson OB (1998). Calculation of the FDG lumped constant by simultaneous measurements of global glucose and FDG metabolism in humans. J Cereb Blood Flow Metab.

[134] Spence AM, Muzi M, Graham MM, O'Sullivan F, Krohn KA, Link JM (1998). Glucose metabolism in human malignant gliomas measured quantitatively with PET, 1-[C-11]glucose and FDG: analysis of the FDG lumped constant. J Nucl Med.

[135] Owen OE, Morgan AP, Kemp HG, Sullivan JM, Herrera MG, Cahill GF (1967). Brain metabolism during fasting. J Clin Invest.

[136] Redies C, Hoffer LJ, Beil C, Marliss EB, Evans AC, Lariviere F (1989). Generalized decrease in brain glucose metabolism during fasting in humans studied by PET. Am J Phys.

[137] Minoshima S, Koeppe RA, Mintun MA, Berger KL, Taylor SF, Frey KA (1993). Automated detection of the intercommissural line for stereotactic localization of functional brain images. J Nucl Med.

[138] Ohnishi T, Hoshi H, Nagamachi S, Jinnouchi S, Flores LG, Futami S (1995). High-resolution SPECT to assess hippocampal perfusion in neuropsychiatric diseases. J Nucl Med.

[139] Kono AK, Ishii K, Sofue K, Miyamoto N, Sakamoto S, Mori E (2007). Fully automatic differential diagnosis system for dementia with Lewy bodies and Alzheimer's disease using FDG-PET and 3D-SSP. Eur J Nucl Med Mol Imaging.

[140] Burdette JH, Minoshima S, Vander Borght T, Tran DD, Kuhl DE (1996). Alzheimer disease: improved visual interpretation of PET images by using three-dimensional stereotaxic surface projections. Radiology..

[141] Lehman VT, Carter RE, Claassen DO, Murphy RC, Lowe V, Petersen RC (2012). Visual assessment versus quantitative three-dimensional stereotactic surface projection fluorodeoxyglucose positron emission tomography for detection of mild cognitive impairment and Alzheimer disease. Clin Nucl Med.

[142] Morbelli S, Brugnolo A, Bossert I, Buschiazzo A, Frisoni GB, Galluzzi S (2015). Visual versus semi-quantitative analysis of 18F-FDG-PET in amnestic MCI: an European Alzheimer's disease consortium (EADC) project. J Alzheimers Dis.

[143] Perani D, Della Rosa PA, Cerami C, Gallivanone F, Fallanca F, Vanoli EG (2014). Validation of an optimized SPM procedure for FDG-PET in dementia diagnosis in a clinical setting. Neuroimage Clin..

[144] Frisoni GB, Bocchetta M, Chételat G, Rabinovici GD, de Leon MJ, Kaye J (2013). Imaging markers for Alzheimer disease: which vs how. Neurology..

[145] Signorini M, Paulesu E, Friston K, Perani D, Colleluori A, Lucignani G (1999). Rapid assessment of regional cerebral metabolic abnormalities in single subjects with quantitative and nonquantitative [18F]FDG PET: a clinical validation of statistical parametric mapping. Neuroimage..

[146] Minoshima S, Frey KA, Koeppe RA, Foster NL, Kuhl DE (1995). A diagnostic approach in Alzheimer's disease using three-dimensional stereotactic surface projections of fluorine-18-FDG PET. J Nucl Med.

[147] Ishii K, Kono AK, Sasaki H, Miyamoto N, Fukuda T, Sakamoto S (2006). Fully automatic diagnostic system for early- and late-onset mild Alzheimer's disease using FDG PET and 3D-SSP. Eur J Nucl Med Mol Imaging.

[148] Wagatsuma K, Sakata M, Ishibashi K, Hirayama A, Kawakami H, Miwa K (2020). Direct comparison of brain [(18)F]FDG images acquired by SiPM-based and PMT-based PET/CT: phantom and clinical studies. EJNMMI Phys.

[149] Garibotto V, Trombella S, Antelmi L, Bosco P, Redolfi A, Tabouret-Viaud C (2020). A comparison of two statistical mapping tools for automated brain FDG-PET analysis in predicting conversion to Alzheimer's disease in subjects with mild cognitive impairment. Curr Alzheimer Res.

[150] Petersen RC, Aisen PS, Beckett LA, Donohue MC, Gamst AC, Harvey DJ (2010). Alzheimer's disease neuroimaging initiative (ADNI): clinical characterization. Neurology..

[151] Caminiti SP, Sala A, Presotto L, Chincarini A, Sestini S, Perani D, et al. Validation of FDG-PET datasets of normal controls for the extraction of SPM-based brain metabolism maps. Eur J Nucl Med Mol Imaging. 2021. 10.1007/s00259-020-05175-1. 10.1007/s00259-020-05175-1 33423088

[152] Thomas BA, Erlandsson K, Modat M, Thurfjell L, Vandenberghe R, Ourselin S (2011). The importance of appropriate partial volume correction for PET quantification in Alzheimer's disease. Eur J Nucl Med Mol Imaging.

[153] Yang J, Hu C, Guo N, Dutta J, Vaina LM, Johnson KA (2017). Partial volume correction for PET quantification and its impact on brain network in Alzheimer's disease. Sci Rep.

[154] López-González FJ, Silva-Rodríguez J, Paredes-Pacheco J, Niñerola-Baizán A, Efthimiou N, Martín-Martín C (2020). Intensity normalization methods in brain FDG-PET quantification. Neuroimage..

[155] Mortensen KN, Gjedde A, Thompson GJ, Herman P, Parent MJ, Rothman DL (2018). Impact of global mean normalization on regional glucose metabolism in the human brain. Neural Plast.

[156] Borghammer P, Jonsdottir KY, Cumming P, Ostergaard K, Vang K, Ashkanian M (2008). Normalization in PET group comparison studies--the importance of a valid reference region. Neuroimage..

[157] Borghammer P (2012). Perfusion and metabolism imaging studies in Parkinson's disease. Dan Med J.

[158] Yakushev I, Landvogt C, Buchholz HG, Fellgiebel A, Hammers A, Scheurich A (2008). Choice of reference area in studies of Alzheimer's disease using positron emission tomography with fluorodeoxyglucose-F18. Psychiatry Res.

[159] Zhang H, Wu P, Ziegler SI, Guan Y, Wang Y, Ge J (2017). Data-driven identification of intensity normalization region based on longitudinal coherency of 18 F-FDG metabolism in the healthy brain. Neuroimage..

[160] Morbelli S, Arbizu J, Booij J, Chen MK, Chetelat G, Cross DJ (2017). The need of standardization and of large clinical studies in an emerging indication of [18 F]FDG PET: the autoimmune encephalitis. Eur J Nucl Med Mol Imaging.

[161] Drzezga A, Arnold S, Minoshima S, Noachtar S, Szecsi J, Winkler P (1999). 18F-FDG PET studies in patients with extratemporal and temporal epilepsy: evaluation of an observer-independent analysis. J Nucl Med.

[162] Busemann Sokole E, Płachcínska A, Britten A, Lyra Georgosopoulou M, Tindale W, Klett R (2010). Routine quality control recommendations for nuclear medicine instrumentation. Eur J Nucl Med Mol Imaging.

[163] Cook GJ, Maisey MN, Fogelman I (1999). Normal variants, artefacts and interpretative pitfalls in PET imaging with 18-fluoro-2-deoxyglucose and carbon-11 methionine. Eur J Nucl Med.

[164] Sala A, Caprioglio C, Santangelo R, Vanoli EG, Iannaccone S, Magnani G (2020). Brain metabolic signatures across the Alzheimer's disease spectrum. Eur J Nucl Med Mol Imaging.

[165] McKeith I, O'Brien J, Walker Z, Tatsch K, Booij J, Darcourt J (2007). Sensitivity and specificity of dopamine transporter imaging with 123I-FP-CIT SPECT in dementia with Lewy bodies: a phase III, multicentre study. Lancet Neurol.

[166] Morbelli S, Esposito G, Arbizu J, Barthel H, Boellaard R, Bohnen NI, et al. EANM practice guideline/SNMMI procedure standard for dopaminergic imaging in Parkinsonian syndromes 1.0. Eur J Nucl Med Mol Imaging. 2020;47:1885–912. 10.1007/s00259-020-04817-8. 10.1007/s00259-020-04817-8 PMC7300075 32388612

[167] Brown RK, Bohnen NI, Wong KK, Minoshima S, Frey KA (2014). Brain PET in suspected dementia: patterns of altered FDG metabolism. Radiographics..

[168] Nestor PJ, Balan K, Cheow HK, Fryer TD, Knibb JA, Xuereb JH (2007). Nuclear imaging can predict pathologic diagnosis in progressive nonfluent aphasia. Neurology..

[169] Nestor PJ, Altomare D, Festari C, Drzezga A, Rivolta J, Walker Z (2018). Clinical utility of FDG-PET for the differential diagnosis among the main forms of dementia. Eur J Nucl Med Mol Imaging.

[170] Whitwell JL, Duffy JR, Strand EA, Machulda MM, Senjem ML, Schwarz CG (2015). Clinical and neuroimaging biomarkers of amyloid-negative logopenic primary progressive aphasia. Brain Lang.

[171] Zorzi G, Cecchin D, Busse C, Perini G, Corbetta M, Cagnin A. Changes of metabolic connectivity in dementia with Lewy bodies with visual hallucinations: a 18F-FDG PET/MR study. Brain Connect. 2021. 10.1089/brain.2020.0988. 10.1089/brain.2020.0988 33757301

[172] Scheltens NME, van der Weijden K, Adriaanse SM, van Assema D, Oomen PP, Krudop WA, et al. Hypometabolism of the posterior cingulate cortex is not restricted to Alzheimer's disease. Neuroimage Clin. 2018;19:625-32. 10.1016/j.nicl.2018.05.024. 10.1016/j.nicl.2018.05.024 PMC6030576 29984170

