# Correction to: EANM procedure guidelines for brain PET imaging using [^18^F]FDG, version 3

**DOI:** 10.1007/s00259-022-05755-3

**Published:** 2022-03-07

**Authors:** Eric Guedj, Andrea Varrone, Ronald Boellaard, Nathalie L. Albert, Henryk Barthel, Bart van Berckel, Matthias Brendel, Diego Cecchin, Ozgul Ekmekcioglu, Valentina Garibotto, Adriaan A. Lammertsma, Ian Law, Iván Peñuelas, Franck Semah, Tatjana Traub-Weidinger, Elsmarieke van de Giessen, Donatienne Van Weehaeghe, Silvia Morbelli

**Affiliations:** 1 APHM, CNRS, Centrale Marseille, Institut Fresnel, Timone Hospital, CERIMED, Nuclear Medicine Department, Aix Marseille Univ, Marseille, France; 2 Service Central de Biophysique et Médecine Nucléaire, Hôpital de la Timone, 264 rue Saint Pierre, 13005 Marseille, France; 3 Department of Clinical Neuroscience, Centre for Psychiatry Research, Karolinska Institutet and Stockholm Healthcare Services, Stockholm, Sweden; 4 Department of Radiology and Nuclear Medicine, Amsterdam UMC, Location VUmc, Amsterdam, The Netherlands; 5 Nuclear Medicine and Molecular Imaging, University Medical Center Groningen, University of Groningen, Groningen, The Netherlands; 6 Department of Nuclear Medicine, Ludwig Maximilians-University of Munich, Munich, Germany; 7 Department of Nuclear Medicine, Leipzig University, Leipzig, Germany; 8 German Centre of Neurodegenerative Diseases (DZNE), Site Munich, Bonn, Germany; 9 Nuclear Medicine Unit, Department of Medicine – DIMED, University of Padua, Padua, Italy; 10 Sisli Hamidiye Etfal Education and Research Hospital, Nuclear Medicine Dept., University of Health Sciences, Istanbul, Turkey; 11 NIMTLab, Faculty of Medicine, Geneva University, Geneva, Switzerland; 12 Division of Nuclear Medicine and Molecular Imaging, Geneva University Hospitals, Geneva, Switzerland; 13 Department of Clinical Physiology, Nuclear Medicine and PET, Rigshospitalet, University of Copenhagen, Copenhagen, Denmark; 14 Department of Nuclear Medicine, Clinica Universidad de Navarra, IdiSNA, University of Navarra, Pamplona, Spain; 15 Nuclear Medicine Department, University Hospital, Lille, France; 16 Division of Nuclear Medicine, Department of Biomedical Imaging and Image‑guided Therapy, Medical University of Vienna, Vienna, Austria; 17 Radiology and Nuclear Medicine, Amsterdam UMC, Location AMC, Meibergdreef 9, Amsterdam, The Netherlands; 18 Nuclear Medicine and Molecular Imaging, University Hospitals Leuven, 3000 Leuven, Belgium; 19 IRCCS Ospedale Policlinico San Martino, Genoa, Italy; 20 Nuclear Medicine Unit, Department of Health Sciences (DISSAL), University of Genoa, Genoa, Italy

**Correction to: Eur J Nucl Med Mol Imaging (2022) 49:632–651**

**https://doi.org/10.1007/s00259-021-05603-w**

The authors regret a typo in the units assessing hyperglycaemia, which should be defined as > 8.9 mmol/L, and not as erroneously mentioned in the published version as > 8.9 nmol/L.

